# ‘Meltdowns’, surveillance and managing emotions; going out with children with autism

**DOI:** 10.1016/j.healthplace.2010.04.012

**Published:** 2010-09

**Authors:** Sara Ryan

**Affiliations:** Health Experiences Research Group, Department of Primary Care, University of Oxford, Old Road Campus, Headington, Oxford OX3 8AH, United Kingdom

**Keywords:** Autism spectrum disorders, Parents, Disabled children, Public places, Interactions

## Abstract

The qualitative study from which the data reported here are taken, explored the experiences, support and information needs of parents of children diagnosed with autism spectrum disorders. 46 parents were interviewed either individually or in couples. Thematic analysis of the data revealed the complexity involved for the parents in taking their children out in public places. The emotion work parents conduct in public places both to make their children more acceptable within the space and to reduce the discomfort that others experience, helps to preserve the orderliness of public places. However, the special competence that parents developed over time also masks their turbulent feelings in public encounters.

## 1 Introduction

Children create disorder in public space when they transgress social, spatial or temporal boundaries (Cloke and Jones, 2005). While such transgressions are often tolerated in very young children, there is an expectation that children are ‘becoming adults’ and will learn the requirements of adulthood (James et al., 1998). Recent work considers the concern surrounding children’s presence in public places and underlines the disquiet associated with the unruly behaviour of children (Valentine, 1996; Matthews and Limb, 1999). For children diagnosed with autism spectrum disorders (ASD), the assimilation of normative ways of behaving is often problematic because of the social, intellectual and sensory difficulties the children may experience. Whilst the difficulties experienced by children with ASD can often be significant, there is often no outward sign of the condition. This can cause particular tensions in public places. Previous research has considered the role parents play in managing public encounters involving children with ASD (for example, Gray, 2002; Ryan, 2005, 2008; Farrugia, 2009), but the emotional management this involves has not been fully examined. Here an analysis of data from interviews with 46 parents of children with ASD focuses on the emotion work parents do in public places and highlights how covert emotion work masks the emotional intensity of public encounters for those present.

## 2 Children in public places

While it has been argued that the traditional authority of parents to discipline and control their children has been eroded (Valentine, 1996; Jamieson and Toynbee, 1989), much literature highlights the significance attached to children becoming competent social actors in public places, and the role parents play in this development (Cahill, 1987; Valentine, 1996; Matthews and Limb, 1999; Philo, 2000; Ryan, 2005). This work suggests that the streets belong to adults and children are permitted into public spaces only when they have been socialised into appropriate adult ways of behaving (Valentine, 1996). This socialisation occurs ‘on the job’ so to speak as children are ‘instructed’, ‘coached’ and ‘primed’ by their caretakers about the ceremonial rules that govern public spaces (Cahill, 1987).

Several layers of disciplinary actions and surveillance, explicit and implicit, underline the position of children in public places. Here we are concerned with the surveillance tactics of those present; their use of stares, glares or comments to inform children and their caretakers that their behaviour is not acceptable. Of course, the children’s behaviour in public places is often not, in and of itself, particularly problematic. Running around, screaming or shouting, cannot be described as criminal activities and yet the collective strength attached to the rules governing behaviour in public places makes such behaviours unacceptable. As Cloke and Jones (2005) suggest, soft criminality is often inferred from the disruption of adult order. It is the outcome of a very rigid form of informal control (Goffman, 1967) operating in public places; ethnomethodologists would argue that the preservation of orderliness matters more than the communication of meaning (Jenkins, 2009).

For interactionists, the self arises in social experiences and is sustained through social interaction (Cahill, 1987). Our ability to put ourselves in the shoes of others and imagine how they perceive us is a powerful tool for facilitating social cohesion in public places though, of course, people’s perceptions may often be distorted and influenced by their life experiences, assumptions and prejudices. Most people internalise social norms and values across the life course and develop strategies and means of maintaining what Goffman (1967) calls ‘poise’ during encounters with others.

Children’s behaviour in public places directly reflects on the perceived competence of their parent or caretaker: “Through smiles, glances and other subtle indications, other adults continually remind children’s public caretakers that their charges’ public behaviour is a reflection of their own moral character” (Cahill, 1987, p. 315). This could explain why parents view children’s public misbehaviour as an “emergency situation” (Brown, 1979). Parents try to present a “good and adequate parent self” by publicly disciplining their children (Brown, 1979) or demonstrating their moral responsibility through apologising to others present or accounting for their children’s behaviour (Ryan, 2008). Social disruption has weighty consequences for offenders, or in the case of (young) children, their caretakers. Social rule breakers, or their caretakers, lose the privilege of civil inattention^1^ and, as a consequence, experience stares and glares from those present (Goffman, 1967).

## 3 Disabled children in public places

Less work has focused on disabled children in public places (Voysey, 1972; Erhmann et al., 1995; Ryan, 2005, 2008); Ryan (2005, 2008) demonstrated how the spatial practices and experiences of mothers of children with learning difficulties were shaped by the reactions of those present and the mothers’ interpretations of, and responses to, those reactions. Mothers shift in the way in which they account for their children’s actions over time as their children’s impairments become more apparent (Ryan, 2008). This shift could be related to the development of a special competence, which enables mothers “to treat as routine occurrences, which embarrass, distress, anger, or otherwise disorientate ‘normal’ members of the public” (Voysey, 1972, p. 88). Voysey (1972) discusses the emotional dimension of this competence, but more generally sociological analysis often downplays emotions (Bridgens, 2009; Williams, 2001).

In the experiences of parents of children with ASD in public spaces, stigma is a consistent theme (Gray, 1993, 2002; Farrugia, 2009). This focus can be explained by the combination of the children’s socially inappropriate behaviour and the lack of outward signs of the condition. The combination of these two factors, Gray (1993, 2002) tells us, leads parents to acquire a courtesy stigma; a spoiled identity through their relationship with their children. I have previously questioned the use of this concept to make sense of the experiences of mothers of disabled children, particularly as parents of disabled children are increasingly understood to accept their children’s diagnoses and incorporate difference within their families (Landsman, 2003; Fisher and Goodley, 2007; Ryan, 2008). Debate remains, however, about the meaning of this normalcy for families (Bridgens, 2009; Lillrank, 2002).

## 4 Public space and emotions

Early theorising about emotions highlights how the feeling and display of emotions are shaped and modified by the interactional rules of conduct (Gross and Stone, 1964; Goffman, 1971; Hothschild, 1979). The interrelationship between emotions, embodiment and spatiality has been increasingly considered (Anderson and Smith, 2001; Davidson, 2004; Thrift, 2004; Colls and Horschelmann, 2009) and there has been a call for a more explicit engagement with emotions as a means of helping us to understand the relationship between people and place (Davidson, 2004).

People become emotionally committed to the code of ceremonial conduct and the transgression of that code, particularly in public places, can result in embarrassment, fear, revulsion, distress and anger (Cahill, 1987). Indeed, Cahill and Eggleton (1994) describe the ‘emotional turbulence’ experienced by wheelchair users in public places. Emotions consistently feature within research focusing on disability and public space (Voysey, 1972; Cahill and Eggleton, 1994; Gray, 2006; Ryan and Raisanen, 2008) albeit often implicitly.

While people may appear reserved and indifferent in public places this appearance is a result of emotion work rather than its absence (Cahill and Eggleton, 1994). Emotion work is the “act of trying to change in degree or quality any emotion or feeling” (Hothschild, 1979, p. 561). Cahill and Eggleton’s (1994) participants used humour to allay anxieties and ease the “dis-ease” of embarrassment, retaining their poise and good humour in the face of frustrating and potentially embarrassing events. They had the “double duty” of managing their own emotions and the emotions of others (Hothschild, 1979). The expression of anger, frustration or indignation at the actions of others in public places can lead to feelings of guilt and embarrassment, hence Hothschild’s (1979) argument that “feeling rules” prescribe anger but ‘expression rules’ forbid its expression; the potential subjective costs and interpersonal risks involved in expressing emotions in public can be significant.

Disruptions in public interactions are a key source of embarrassment, so it is not surprising that accounts of parents of children with ASD describe being embarrassed in public places (Gray, 1993, 2002). The strength of embarrassment is of fundamental social and moral significance (Williams, 2001) and is closely related to shame. Parents of children with ASD are effectively going out with regular rule breakers and so consistently experience the breaching of taken for granted assumptions (Garfinkel, 1967). Given the lack of outward signs of ASD, parents often appear to be incompetent parents, rather than as parents of children with particular social and communication disorders. Here I unravel the complex layers involved in public interactions for parents of children with ASD and demonstrate how, while these encounters remain emotionally charged for parents, their actions work to reduce the emotional engagement of others present.

## 5 Methodology

The broader UK based study from which these data come, explored the experiences, support and information needs of people diagnosed with ASD and parents of children diagnosed ASD. The methods have also been described elsewhere (Ryan and Runswick-Cole, 2008; Ryan and Raisanen, 2008). Qualitative interview methods were used to understand how participants made sense of their lives and their children’s diagnosis, the issues or life events of significance to them and how they negotiated the social world. While the overall study included people on the autism spectrum, the analysis here focuses upon the parents.

The sample was recruited through support groups, newsletters, online communities, special schools and local authority parent co-ordinators. A maximum variation approach (Coynem, 1997) was taken to include a range of participants of different ages, social class, geographical location, the ability/disability of the child with an ASD, and the number of siblings within the family. Forty eight parents, who were predominantly White British but included participants who were White other, Black British and mixed, aged between 18 and 80, were interviewed; 7 couples, 5 fathers and 29 mothers. Just under half of the women and one man were full time carers, the remainder of participants had a variety of occupations including teacher, health worker, student, dinner lady, mechanic, insurance broker and company manager. The children, aged between 3 and 53 were all on the autism spectrum with diagnoses of autism, Asperger syndrome and atypical autism. Seven families had two or more children on the autism spectrum. Two parents who were diagnosed with ASD were excluded from this analysis as they did not discuss their experiences of going out in public in relation to their children, but in relation to themselves.

In-depth interviews were conducted largely at participants’ homes (two in office settings). The interviews were in two parts: first, parents were asked “Can you tell me about your experiences with your son/daughter?” This question prompted lengthy uninterrupted narratives, which were largely chronological and lasted between 20 min and just under two hours. The second part of the interview was semi-structured and questions included “What sort of impact do you think these experiences have had on you?” “Can you describe your dealings with health professionals?”.

Interviews lasted between one and three hours and were recorded and transcribed verbatim. The data were analysed electronically using a thematic approach with the organisational support of Nvivo. Data was open coded into different categories such as early signs, getting a diagnosis, everyday life, going out and perceptions of cause. A constant comparative method was used to develop and refine the theoretical categories (Seale, 1999). Categories were repeatedly compared and integrated and several key themes emerged. Given my previous research, difficulties associated with going out was an anticipated theme and most participants spontaneously discussed their going out experiences in the first part of the interview. Those participants who did not, either discussed going out when asked to talk about their everyday lives and two participants were directly asked: “Can you tell me about your experiences of going out in public with your son/daughter?” The emotional labour associated with going out was an emergent theme; participants were not explicitly asked about how they felt going out in public places.

Further analyses and extracts from the interviews can be seen at 〈www.healthtalkonline.org〉.

## 6 Findings

The main problems facing participants in public places were the unpredictability of the behaviour of their children and of the responses of those present.

### 6.1 Unpredictable children

All parents gave examples of behaviour by their children that suggested that they were acutely aware of what is, and what is not, socially acceptable behaviour. Martin, for example, reflected on his experiences before his son went to residential school aged 14:

So it is things like that, you have got to adapt your life everywhere. You know. I think what it is for us as a family [um] we used to go out a lot. We sort of stopped going out a lot. You feel like you don’t want to. … It is an embarrassment. You think ‘Am I going to get embarrassed here?’ But then you think, you have got to overcome that. That is what you have got to overcome, because the child has got to have a normal life. So we just tried to include him like in our normal family things, you know, but there would be certain things you couldn’t do, i.e. we couldn’t go to the pictures because he didn’t like the cinema. He doesn’t like the dark and so he would disrupt it totally so we would end up having to try and get someone to babysit him while we went to the pictures. So it is things like that. You know it does hurt. (Martin)

Martin describes his embarrassment in public with his son, but also the hurt he feels about not being able to sustain “normal” family life. This hurt was articulated by several parents who missed the spontaneity they perceived other families to have when it came to going out:

Just a simple thing like going into the shop and buying a pint of milk can present itself to be a big issue and that gets you down quite often. (Jennifer, son aged 11)

The prescribed goal of ‘normal life’ is common in accounts of parents of disabled children (Bridgens, 2009). Other participants similarly described a moral imperative to overcome the embarrassment and disruption to try to achieve normal family life, although parents of younger children were more likely to describe still actively trying to achieve this, than parents of older children as the following extract illustrates:

We go on a ferry to France every year and you know it is stressful but you know you have got to sort of keep going doing things and you know try and get them used to … you know they are frightened of a lot of things and the world is scary for them but you have got to try and gently do it, you know get them out, get them doing things [um]. (Angela, 2 children aged 5 and 3)

Many parents of older children reduced the amount they went out in public to avoid the difficulties inherent in these outings:

But … yes, so the less confrontation you can have the better all round and I don’t mean that you have to let them do what they want and [um] I must say I am quite bad because, in a way, because it is very hard, with family and going to other people’s houses and that. So Tom and I do, we do keep to ourselves a lot. (Carol, son aged 11)

Most participants described preparing their children in advance of going out (by explaining where they were going, for example), but they could not always predict how their children would respond to particular environments or be in a position to always prepare, as the following extracts illustrate:

You have got to be continually on your guard, when you say, “No, we are not going into the shop, and no, if we go into the shop you can’t buy anything.” And you have always got to always be on your guard to tell him that beforehand. And generally if you lay the ground rules before you go into a risky situation it is okay, but there are times when you are busy, there is a lot going on and you don’t explain everything to him … (Martin, son 8)

When she is naughty, you look at Mandy when she is like now, when she is walking along, no one would think anything was wrong but all of a sudden in the supermarket she will just have a hissy fit and you get the dirty looks, and you get the ‘tch haa’ because these people don’t know that that is what they are, that is what they do [um] and there is no way that you can stop that because it is just spontaneous, you just don’t sort of really know … I sort of see a few signs, you might be able to predict it is going to happen, but not all the time. (Tina, daughter 18)

These two extracts highlight how uncertainty and unpredictability are an integral part of going out despite efforts to prepare, or even when the children become adults.

That children with ASD can be disruptive is well established (Gray, 1993, 2002; Farrugia, 2009) but little attention has been paid to why that might be. In the current study, many participants linked their children’s disruptive behaviours to sensory issues and the difficulties children with ASD have dealing with different settings. Children went into “meltdown” not as wilful displays of bad behaviour but as intense responses to overwhelming situations. Lights, unusual noises, darkness, crowds, queues, smells, unfamiliar places and people can all bewilder and overwhelm children with ASD in public spaces:

There was a lot of music, there was a lot of chattering in the shop, it was very hot. And those three things in combination completely overwhelmed him. He couldn’t cope with it. (Nina, son aged 6)

It was things like taking him into public loos and if anybody turned the hand drier on he went berserk because the noise must have hurt his ears, but you just don’t know what’s the matter. You are distressed because they are distressed. (Jane, son aged 13)

Participants were managing their children’s ability to cope with different settings at the same time as dealing with the responses of others present and were attuned to the sensory dimensions to public spaces that their children found difficult. The empathetic understanding most parents demonstrated of their children’s sensory sensitivities offers an alternative (and so far overlooked) interpretation of the experiences of parents of children with ASD in public places. An emphasis on embarrassment (Gray, 1993) highlights the parent-other interaction rather than the emotional experience of the child and the accompanying impact on the parents. As Jane suggests, in the above extract, it is distressing to witness the distress of your children.

What is also important is the difficulty people not familiar with ASD appear to have in understanding how intensely children can be affected by sensory factors. This lack of understanding can extend to close family, as the following extract illustrates:

We have got a christening coming up in a couple of weeks. It is my brother’s son’s christening so it is immediate family but there is no way Guy could go you know, no way and even close family, I think, struggle with how difficult it is for Guy to do something like that. (Karen, son aged 8)

Participants had to manage not only the unpredictability of their children’s behaviour but also the responses of others to that behaviour.

### 6.2 Unpredictable others

The emotional turbulence parents experienced was very clear in their accounts. This was related not only to the possible distress of the children but for a few parents, also the internalised concern they themselves often had about what others think of them:

The hardest thing, one of the hardest things I find is other people. You know that is the thing I am always bothered about. I know it is a problem more in my head and other people just say “I don’t care what other people think”, but you know from when he was little and he used to scream and head bang and people used to stare in shops […] and even now, you know when he goes out in his slippers he looks different and going into our local post office that we go into almost every day, and they say, “Hello.” And he doesn’t say hello back. I feel uncomfortable with that. (Karen, son aged 9)

Karen highlights her commitment to the ceremonial rules and how conscious she is that her son is breaching them. Similarly, Laura articulates a tension between her protectiveness for her son, combined with a feeling that perhaps he could behave differently:

And I think I find it hard with him in public [um] because I am just on edge, you know, and I jump to his defence and at the same time, I want him to behave like I know he can behave [um]… (Laura, son aged 11)

While some participants talked about difficulties around safety when they took their children out—in terms of them running off or having little or no awareness of road or stranger safety—it was the responses of others present, and parents’ implicit understanding of the reasons behind those responses (whether they judged them to be fair or not), that they found particularly difficult to deal with. The above extracts again underline participants’ awareness of the rule breaching their children do in public places.

The emotional intensity and internal conflicts involved in managing public encounters is illustrated by the following extract in which Nina describes her response to a supermarket cashier:

And that stirred up a whole load of emotions for me. [um] I was torn three ways. Firstly it was to say to her, “Who do you think you are? You don’t know me. You don’t know my son. How dare you judge me? And how dare you bring it to the attention of your friends.” That was my first reaction. My second reaction was to [um] ignore her completely, ignore it completely, shut it out, pretend it wasn’t happening and just deal with Tim. My third reaction was to [um] say to her, “My son is autistic,” and approach it in a way that was, you know, please understand. And actually of those three I didn’t know which way to go. So I just stayed quiet, dealt with Tim and got out the shop. (Nina, son aged 6)

No guidelines exist in parenting literature to help parents of children with ASD manage public encounters although the internet offers spaces where parents can discuss their experiences (see, for example, www.asdfriendly.org). The experiences described here are beyond the scope of most people’s experiences of going out and Nina’s account captures the tension between feeling rules and expression rules. She describes being angry and upset, but retains her emotional poise and does not express her anger. This is a common response among disabled people in public places more generally (Cahill and Eggleton, 1994) and underlines commitment to the preservation of orderliness rather than establishing meaning within interactions (Jenkins, 2009). There are subjective costs in publicly expressing anger and it was clear that parents learned over time ways of managing public encounters, which were less emotionally fraught for those present, enabling them to retain their poise (Goffman, 1967). I will return to this in a later section.

### 6.2.1 Stares, glares and NAS^2^ cards

The majority of parents described experiencing looks, stares or glares when they were out with their children. This again is a consistent theme in existing studies. These looks, which indicate that the principle of civil inattention has been withdrawn, were interpreted in different ways by participants. The first was a form of surveillance of their parenting skills and this was common among participants with younger children:

But it is quite difficult when you are out and you are out in the supermarket because you have got you know, the added pressure of everybody looking at you [um] because what they see is. I mean, you can’t blame them, he looks completely normal. There is nothing different about the way he looks. And he wants a DVD and he is not going to get one and so he kicks off. So the general public what they are seeing is a spoilt brat whose mum obviously gives into him all the time. So we get a lot of sort of tutting and “Tut, if that were my child … ” kind of thing. (Angela, 2 children 5 and 3)

Parents experiencing this type of stare or comment articulated a spoiled identity; they felt that people thought they were poor parents. Such judgements could be counteracted by disclosing the child’s autism, thereby restoring the moral identity of the parent. The ‘spoiled identity’ parents experienced, was the “poor parent” identity as opposed to the parent of a child with ASD (see also, Ryan, 2008; Farrugia, 2009). One of the strategies several parents used to resolve this was to disclose their children’s autism either verbally or through the use of badges, labelled t-shirts or, most commonly, handing out cards from the NAS:

And we have got these … Because I am a member of the National Autistic Society, so we have these little cards that I got on the website and it explains about it says this young person has autism and explains that there could be little outbursts and please be patient and understanding with us, you know. (Catherine, son aged 10)

Those two incidences I had actually prompted me to get some of those cards because they are just little business cards and they just simply say across the card, ‘This young person has autism.’ And then it just gives you a couple of bullet points of what autism is and it says if you want to know more please contact the NAS or go to the website and for us, or for me they just stopped me getting into that, do I shout at this person for being so rude, or do I get upset? How do I deal with this? (Nina, son aged 6)

For these parents, these cards solved the emotional dilemma of trying to manage public encounters. As Nina said, difficult encounters can be managed in various ways and handing over a card involves less emotional engagement. In the following extract, her husband Pete describes handing a card to a man who complained about their son kicking his seat in the cinema:

I am quite a fiery bloke myself so, I mean for me to stand out and actually pull out my wallet and give him a card was a major achievement on my part as well as the guys and my son’s and everything else and to be fair it took a real lot of doing because it was really quite a stressful situation. I mean had things, had he raised his voice, then I probably would have raised my voice back… (Pete, son aged 6)

This extract highlights how the cards can be used as a subtle way of shifting the attention back to the “starer” who becomes the person in the spotlight once they have the card. The concept of audience role prominence (Lofland, 1989) suggests that people prefer to be part of the crowd rather than singled out in public encounters and Pete described how the person who had complained about his son left the cinema looking “shamed by what he had done”. But clearly parents chose when to hand out a card or when to do something different. Public encounters are often too populated to distribute cards to everyone present.

In addition to cards, one parent feigned sign language to alert people present that something was different about her son, while another customised clothing:

And a few years ago as well when we went on holiday, because I got sick of people sort of like I say looking at us and gawping and sort of pointing the finger you know. I had some t-shirts printed. All different colours, red, green, blue, white, all with different coloured writing on to match whatever he was wearing, different outfits. And it just simply said on it. “I am not naughty. I am autistic.” And do you know the amount of people that come up to us because of those t-shirts …(Catherine, son aged 10)

Yes and you know, he’ll bang things maybe, as he is going past and people, you know, look at him, and clearly are a bit worried. So I frequently will say something or I will talk to him—Roger you do this as well in public—talk to him in such a way that people around realise that something is not quite as it should be and if they know that you are with him, then people are reassured and it stops any problems arising. (Georgina, son 28)

Again, these strategies reduce the level of emotional engagement that verbal disclosure entails including who to tell, what to tell, how much to tell and when to tell. As existing research highlights, disclosure (or explanatory) narratives (Jenks, 2005) can be complex and their use can vary both temporally and spatially. Again, the emotional dimension to disclosure narratives is a significant factor for parents:

When people stop and stare in the street or he is having a tantrum and everybody is looking at you thinking, “Can’t you control that child?” and you just, you get really upset because it is not his fault that he is being like that. And not a lot of people understand what autism is. If you explain to them, they do understand but it can take a lot of explanation and when you are very tired and you are coping with a child like he is, you do get very fraught and your emotions… and so sometimes you just can’t be bothered to tell everybody and you just think let them think it is a naughty child. (Mandy, son 3)

Most parents did not view their children as having a spoiled identity once their autistic identity was established. Indeed, the example above of printing t-shirts suggests that passing is not something that some parents are as concerned about. The children are autistic and that is an integral part of their identity. Only one father (one of the older participants in the sample) hesitated to label his son autistic, though the concern was particularly focused on disclosing autism in front of his son, rather than to other people:

There is one thing I do find quite difficult. [um] And that is the decision whether or not to say to a stranger, that [um] [er] this youngish man with me, has autism… But a big part of the problem is a feeling of some embarrassment in front of Geoff for sort of labelling him in his presence [um] as being autistic. (Roger, son aged 28)

For the remaining parents, the child’s identity as a “normal” child was the spoiled identity. Perceptions of the children as “normal” led to misinterpretations of the children’s behaviour and, as a consequence, the parents’ competence in bringing up children. Disclosure was often effective in restoring the moral identity of the parents. This is consistent with previous research (Gray, 1993, 2002; Ryan, 2005; Farrugia, 2009).

The second interpretation of stares and looks was that the gaze was directed at the child and indicated a negative evaluation of the child:

I suppose I used to be quite embarrassed that people were looking at me and I used to think when John was having a paddy in a shop or something I used to think ‘oh god, people are going to think he is horrible’ and he is not and I used to be saying all the time, “Oh I am sorry, my little boy is autistic, and he can’t help it”. (Trish, 2 sons aged 8 and 6)

Parents felt protective of their children and did not want them to be judged by inappropriate standards. Another mother described negative responses towards her son as upsetting because she feared her son would not be accepted in public places as he grew older. Again, disclosure of ASD could resolve this problem.

The third interpretation was that the gaze was an indication of curiosity. People looked because they detected something different about the child’s behaviour. For example:

Like … and all the vegetables he would pick up and want to eat them and you think yes let him have a carrot and he would eat as you go round but he would pick up boxes and it used to be really stressful and other people would look at him and you could see them looking at him and thinking ‘oh there is something not quite right there’. (Stella, son aged 14)

It can be very wearing to be stared at when my daughters act inappropriately, but I guess that people are just curious. Mostly, I’ve been impressed with peoples’ kindness, once they understand that my children have autism. (Susan, daughters 14 and 16)

Again, being looked at is an indication that the children, and their parents, have lost the right to civil inattention and so people feel they have a right to stare and glare at them (Goffman, 1967, 1971). But whatever the motivation behind the stare, it discomfited and upset parents:

Simon: There is nothing … like Catherine said …. There is nothing worse than people gawping at you when your kid is having a fit and you know, you just …Catherine: And you just feel like bursting into tears because you do. (son aged 10)

Some parents reflected on a lessening of the emotional intensity they experienced in public encounters over time:

I am now able to deal with, even you know, the embarrassment of being in a shop and him having an outburst. We carry the little cards around with me and sometimes I have to give them out to people. And sometimes I don’t. […] But over the last few years the ones that will now sort of throw it on the floor, you grow very thick skin and there are sometimes when you turn round and tell them what you really think and other times when you are able just to walk away because you know that it is basically ignorance and you are not going to get anywhere with them. So that has become easier. (Madge, son aged 10)

But [um] I quite enjoy talking about the boys and why they are the way they are now. I am not fazed. I am proud of them. I’m very proud of them. But it did take quite a while for this feeling of having to apologise. And I thought why should I? They are just my boys. (Trish, 2 sons aged 8 and 6)

Participants appear to become resilient and self confident in their dealings with the public through experience over time. Similarly, Nina describes a situation when she disclosed her son’s autism to the person serving her in a busy shoe shop:

I said it very quietly because I didn’t feel that I owed anybody else an explanation. If they wanted to judge me as a bad mother that is fine. I have got broad shoulders. If they wanted to judge my son as a bad son that is fine. He is my son, he is not theirs. (Nina, son aged 6)

Nina’s course of action in this setting is in contrast to other experiences she recalled during the interview. It was clear from the data that public spaces were experienced as multi-faceted; each setting had unique social and spatial characteristics.

## 7 Discussion and conclusion

This analysis demonstrates the complex emotional labour parents undertake when they go out in public with their children with ASD. They try to manage the unpredictability of their children’s behaviour, their children’s distress and the responses of others present. Each setting has a unique combination of social, spatial (and temporal) characteristics and the outcome, in terms of the children’s behaviour, the responses of others and the parents’ management of the encounter remain uncertain.

That most participants talked about these experiences as significant (bringing them up unprompted during the interviews) underlines their importance for them. A further emotional layer for parents is created by the lack of outward signs of ASD; disapproval from those present is often based on inappropriate judgements about the children (and parents). This analysis suggests that disclosing the children’s autistic identity is a largely effective strategy, replacing the spoiled identities of incompetent parent and badly behaved child. However, disclosure is not always practical in public settings where there is a constant flow of people. The use of cards or other symbols to identify the children as autistic reduces the emotional engagement for the parent.

That parents often want to establish their children’s identity as autistic children in public places suggests that they don’t perceive the diagnosis of ASD to be a spoiled identity or not as bad as being perceived as bad parents. This supports the findings of previous research (Ryan, 2008). Of course, for others present, it may be. For most parents, being autistic is not a pejorative identity but an identity complicated by the lack of visibility and awareness of ASD. Only one parent described feeling uncomfortable about disclosing the label of autism in front of his son. The remainder did not express concern, discomfort or shame about their children’s autistic identity.

Consistent with existing research, (Cahill and Eggleton, 1994), parents’ ‘surface acting’ (Hothschild, 1979) facilitates the smooth running of public encounters. They undertake the double duty of managing their own emotions as well as the emotions of others, demonstrating their commitment to the ceremonial rules governing public places, despite the emotional cost to them. This local work by the parents, and the way in which they retain poise, rather than expressing their feelings about the responses of others towards their children or themselves, leaves the general public unaware of the emotional turmoil experienced by the parents.

The lack of an empathetic understanding by most people outside of (very) close family, of the ways in which children with ASD experience some public places, creates barriers to their acceptance in public places. Parents demonstrated an empathetic understanding of their children’s sensory sensitivities; a dimension, which has been overlooked within existing research. A greater understanding of the experience of ASD may develop with the increasing publication of autobiographical accounts of people with ASD (see, for example, Williams, 1995, 1998; Miller, 2003) and studies exploring the subjective experiences of people with ASD (Davidson, 2007; Ryan and Raisanen, 2008). A greater awareness of the emotional complexity of public encounters for parents of children with ASD together with a greater understanding of the children’s behaviour could lead to more tolerance for unusual behaviours in public places.

Many parents described how they had changed their approach to public encounters over time. They developed a deeper understanding of the ceremonial rules that facilitate a working consensus in public. While the settings remained unpredictable, parents learned ways of dealing with difficult situations, although these were not always effective. Managing the constant rule breaching by their children led parents to occupy an extraordinary space; one occupied by other groups who deviate from the narrow rules governing behaviour in public space (see, for example, Cahill, 1987; Lenny and Sercombe, 2002). In effect, parents gained sociological insights into public order and disorder and learned ways of managing situations that were unfamiliar to them before having a child with ASD. They developed a special competence (Voysey, 1972, 1975) but this competence could involve some difficult learning experiences along the way. The description of public interactions as the ‘hardest thing’ to deal with highlights the centrality of public encounters to our everyday lives. The combination the intense distress public places can create for children with ASD, the effect of the disciplinary gaze and lack of understanding often shown by people present and the emotional turbulence this creates for the parents, led parents to take their children out less. This is a matter of concern, not only for the children, their parents and other family members, but also for society more generally.

## 8 Directions for future studies

A limitation of this study is that participants are reporting their experiences rather than the experiences being observed first hand. We can’t know what others present were thinking or the intention behind their responses to the parents and their children. We also do not know what the children think about being openly labelled as autistic. These gaps warrants further research, particularly as we are now gaining a fuller understanding of the experiences of parents through this study and earlier research (Ryan, 2005, 2008; Gray, 2002). Understanding how the children and others present interpret these public encounters is a complicated area to explore but could offer greater sociological insights into the relevance of orderliness in public places. A further direction for study, and one that is often overlooked in research in this area, is an exploration of how the diagnosis of ASD intersects with other facets of family practice and identity such as age, gender, ethnicity and social class.

## Footnotes

1 Civil inattention is a principle of social interaction in public places whereby people glance at each other to show they are aware of each other’s presence, but the glance is fleeting so that people do not feel they are a target of special curiosity (Goffman, 1967, p. 84)

2 The National Autistic Society (www.nas.org) is the national autism support organisation in the UK.

## References

[bib1] Anderson. K., Smith S. (2001). Editorial: emotional geographies. Transactions of the Institute of British Geographers.

[bib2] Bridgens R. (2009). Disability and being ‘normal’: a response to McLaughlin and Goodley. Sociology.

[bib3] Brown W.B. (1979). Parents’ discipline of children in public places. The Family Coordinator.

[bib4] Cahill S.E. (1987). Children and civility: ceremonial deviance and the acquisition of ritual competence. Social Psychology Quarterly.

[bib5] Cahill S.E., Eggleton R. (1994). Managing emotions in public: the case of wheelchair users. Social Psychology Quarterly.

[bib6] Colls R., Horschelmann K. (2009). The geographies of children’s and young people’s bodies. Children’s Geographies.

[bib7] Cloke P., Jones O. (2005). Unclaimed territory: childhood and disordered space(s). Social and Cultural Geography.

[bib9] Coynem M.J. (1997). Sampling in qualitative research. Purposeful and theoretical sampling; merging or clear boundaries?. Journal of Advanced Nursing.

[bib10] Davidson J. (2004). Embodying emotion sensing space: introduction to emotional geographies. Social and Cultural Geography.

[bib11] Davidson J. (2007). ‘In a world of her own…’ re-presenting alienation and emotion in the lives and writings of women with autism. Gender, Place and Culture.

[bib13] Erhmann L.C., Aeschleman S.R., Svanum S. (1995). Parental reports of community activity patterns: a comparison between young children with disabilities and their non-disabled peers. Research in Developmental Disabilities.

[bib14] Farrugia D. (2009). Exploring stigma: medical knowledge and the stigmatisation of children diagnosed with autism spectrum disorders. Sociology of Health and Illness.

[bib15] Garfinkel H. (1967). Studies in Ethnomethodology.

[bib16] Goffman E. (1967). Interaction ritual: essays on face-to-face interaction.

[bib17] Goffman E. (1971). Relations in public: microstudies of the public order.

[bib18] Fisher P., Goodley D. (2007). The linear medical model of disability: mothers of disabled babies resist with counter-narratives. Sociology of Health and Illness.

[bib19] Gray D.E. (1993). Perceptions of stigma: the parents of autistic children. Sociology of Health and Illness.

[bib20] Gray D.E. (2002). Everybody just freezes. Everybody is just embarrassed: felt and enacted stigma among parents of children with high functioning autism. Sociology of Health and Illness.

[bib21] Gray D.E. (2006). Coping over time: the parents of children with autism. Journal of Intellectual Disability Research.

[bib22] Gross F., Stone G.P. (1964). Embarrassment and the analysis of role requirements. The American Journal of Sociology.

[bib49] James O., Jenks C., Prout A. (1998). Theorising Childhood.

[bib23] Jamieson L., Toynbee P., Corr H., Jamieson L. (1989). Shifting patterns of parental authority, 1900–1980. The Politics of Everyday Life..

[bib24] Jenkins K.N. (2009). Studies in and of ethnomethodology: Garfinkel and his ethnomethodological ‘bastards’. Part 2. Sociology.

[bib25] Jenks E.B. (2005). Explaining disability: parents’ stories of raising children with visual impairment in a sighted world. Journal of Contemporary Ethnography.

[bib27] Hothschild A. (1979). Emotion work, feeling rules and social structure. American Journal of Sociology.

[bib28] Landsman G. (2003). Emplotting children’s lives: developmental delay vs. disability. Social Science and Medicine.

[bib29] Lenny M., Sercombe H. (2002). Did you see that guy in the wheelchair down the pub? Interactions across difference in a public place. Disability and Society.

[bib30] Lillrank A. (2002). The tension between overt talk and covert emotions in illness narratives: transition from clinician to researcher. Culture, Medicine and Psychiatry.

[bib31] Lofland L. (1989). Social life in the public realm: a review. Journal of Contemporary Ethnography.

[bib32] Matthews H., Limb M. (1999). Defining an agenda for the geography of children: review and prospect. Progress in Human Geography.

[bib33] Miller, J.K., 2003. Women from another planet: Our lives in the Universe of Autism. Dancing Minds, Bloomington, IN.

[bib35] Philo C. (2000). ‘The corner-stones of my world’: editorial introduction to a special issue on spaces of childhood. Childhood.

[bib36] Ryan S. (2005). “People don’t do odd, do they?” Mothers making sense of the reactions of others towards their learning disabled children in public places. Children’s Geographies.

[bib37] Ryan S. (2008). “I used to worry about what other people thought but now I just think … well I don’t care”: Shifting accounts of learning difficulties in public places. Health and Place.

[bib38] Ryan S., Raisanen U. (2008). “It’s like you are just a spectator in this thing”: experiencing social life the ‘aspie’ way. Emotion. Space and Society.

[bib39] Ryan S., Runswick-Cole C. (2008). Repositioning mothers: mothers, disabled children and disability studies. Disability and Society.

[bib40] Seale C. (1999). The Quality of Qualitative Research.

[bib41] Thrift N. (2004). Intensities of feeling: towards a spatial politics of affect. Geografiska Annaler..

[bib43] Valentine G. (1996). Angels and devils: moral landscapes of childhood. Environment and Planning D: Society and Space.

[bib44] Voysey M. (1972). Impression management by parents with disabled children. Journal of Health and Social Behaviour.

[bib45] Voysey M. (1975). A Constant Burden.

[bib46] Williams D. (1998). Like Colour to the Blind: Soul Searching and Soul Finding.

[bib47] Williams D. (1995). Nobody, Nowhere.

[bib48] Williams S. (2001). Emotion and Social Theory.

